# t(8;9)(p22;p24)/PCM1-JAK2 Activates SOCS2 and SOCS3 via STAT5

**DOI:** 10.1371/journal.pone.0053767

**Published:** 2013-01-23

**Authors:** Stefan Ehrentraut, Stefan Nagel, Michaela E. Scherr, Björn Schneider, Hilmar Quentmeier, Robert Geffers, Maren Kaufmann, Corinna Meyer, Monika Prochorec-Sobieszek, Rhett P. Ketterling, Ryan A. Knudson, Andrew L. Feldman, Marshall E. Kadin, Hans G. Drexler, Roderick A. F. MacLeod

**Affiliations:** 1 Leibniz Institute, DSMZ - German Collection of Microorganisms and Cell Cultures, Department of Human and Animal Cell Cultures, Braunschweig, Germany; 2 Department of Hematology, Hemostasis, Oncology, and Stem Cell Transplantation, Medical School Hannover, Hannover, Germany; 3 University of Rostock, Institute of Pathology and Molecular Pathology, Rostock, Germany; 4 Department of Genome Analysis, HZI-Helmholtz Centre for Infection Research, Braunschweig, Germany; 5 Department of Diagnostic Hematology, Institute of Hematology and Transfusion Medicine, Warsaw, Poland; 6 College of Medicine, Department of Laboratory Medicine and Pathology, Mayo Clinic, Rochester, Minnesota, United States of America; 7 Boston University School of Medicine, Department of Dermatology and Skin Surgery, Roger Williams Medical Center, Providence, Rhode Island, United States of America; University of Navarra, Center for Applied Medical Research, Spain

## Abstract

Fusions of the tyrosine kinase domain of JAK2 with multiple partners occur in leukemia/lymphoma where they reportedly promote JAK2-oligomerization and autonomous signalling, Affected entities are promising candidates for therapy with JAK2 signalling inhibitors. While JAK2-translocations occur in myeloid, B-cell and T-cell lymphoid neoplasms, our findings suggest their incidence among the last group is low. Here we describe the genomic, transcriptional and signalling characteristics of PCM1-JAK2 formed by t(8;9)(p22;p24) in a trio of cell lines established at indolent (MAC-1) and aggressive (MAC-2A/2B) phases of a cutaneous T-cell lymphoma (CTCL). To investigate signalling, PCM1-JAK2 was subjected to lentiviral knockdown which inhibited 7 top upregulated genes in t(8;9) cells, notably SOCS2/3. SOCS3, but not SOCS2, was also upregulated in a chronic eosinophilic leukemia bearing PCM1-JAK2, highlighting its role as a central signalling target of JAK2 translocation neoplasia. Conversely, expression of GATA3, a key T-cell developmental gene silenced in aggressive lymphoma cells, was partially restored by PCM1-JAK2 knockdown. Treatment with a selective JAK2 inhibitor (TG101348) to which MAC-1/2A/2B cells were conspicuously sensitive confirmed knockdown results and highlighted JAK2 as the active moiety. PCM1-JAK2 signalling required pSTAT5, supporting a general paradigm of STAT5 activation by JAK2 alterations in lymphoid malignancies. MAC-1/2A/2B - the first JAK2–translocation leukemia/lymphoma cell lines described - display conspicuous JAK/STAT signalling accompanied by T-cell developmental and autoimmune disease gene expression signatures, confirming their fitness as CTCL disease models. Our data support further investigation of SOCS2/3 as signalling effectors, prognostic indicators and potential therapeutic targets in cancers with JAK2 rearrangements.

## Introduction

Janus (tyrosine) kinases (JAK) are deregulated in leukemia/lymphoma by copy number alterations (CNA), mutations and chromosomal translocations. While mutations affecting JAK2 (JAK2mu) have been widely investigated in recent years, the rarer yet more structurally and clinically varied JAK2 translocation neoplasms remain weakly characterized. The advent of small molecule inhibitors has highlighted JAKs and their effectors as potential therapeutic targets and JAK2 translocation malignancies are prime candidates for selective inhibitor treatments which among JAK2mu neoplasia have been rewarded with but moderate success hitherto [1].

**Figure 1:**
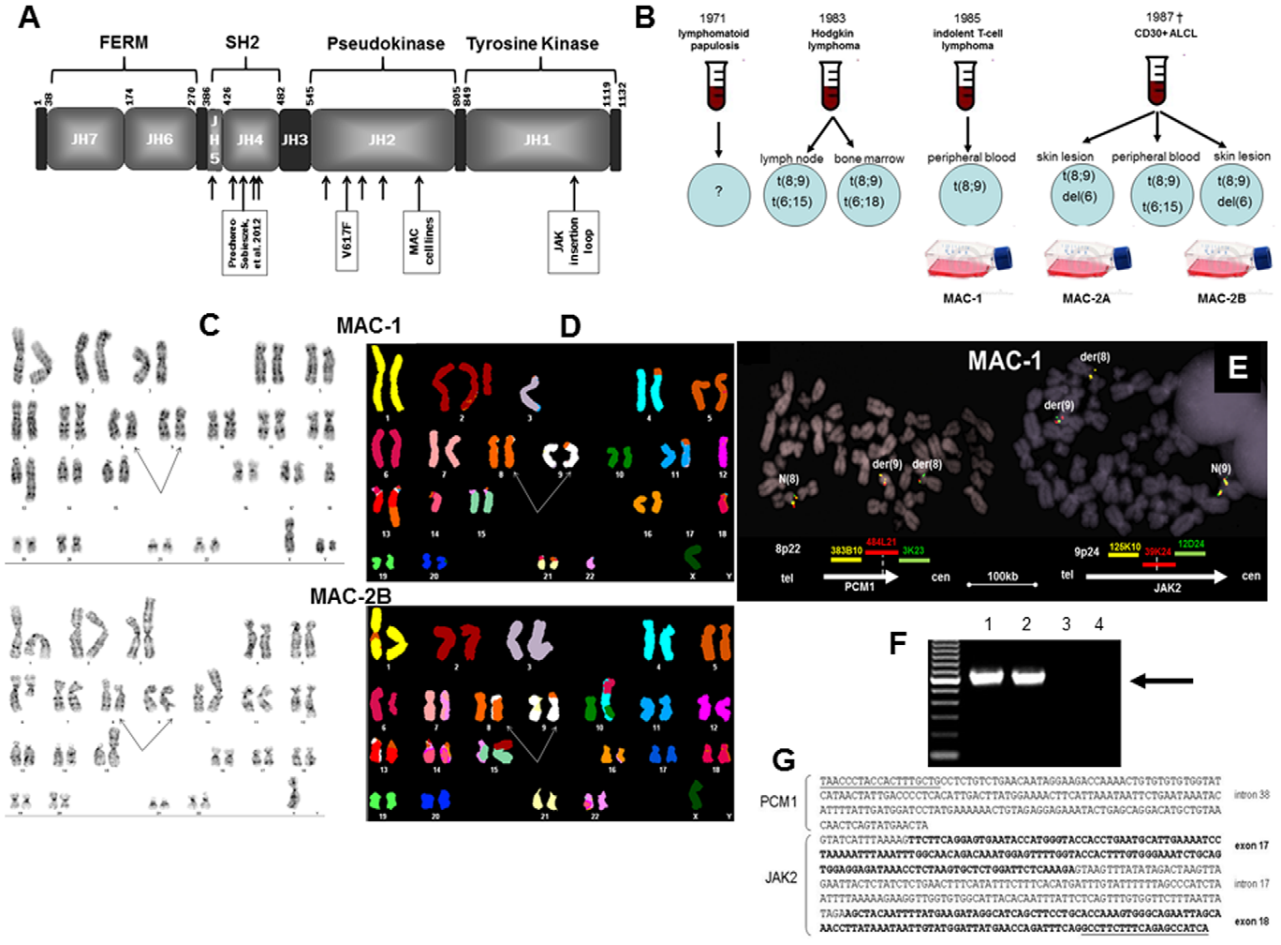
Genomic analysis. **A**: Depicts the domain structure of JAK2 (amino acid numbering based on Chen et al. [2]. Arrows show breakpoints reported in MAC cells and t(8;9) patients. **B**: Diagrammatic history of t(8;9) observed in the donor patient and derived cell lines at various times throughout the course of disease. t(8;9) was accompanied by various chromosome 6 rearrangements. Analyses were based on classical methods only. **C**: G-banding, and **D**: SKY analyses of MAC-1 and MAC-2B cells which were the most similar to MAC-2A (not depicted). Arrows show t(8;9). **E**: Shows FISH using BAC clones flanking and straddling PCM1 (left) and JAK2 (right): Note breaks (split signals) within RP11-484L21 (PCM1) and RP11-39K24 (JAK2). **F**: Shows gPCR for PCM1-JAK2 unique to MAC-1/2A/2B, and **G**: sequencing of the genomic PCR product of t(8;9) breakpoints in PCM1 and JAK2. Abbreviation: NTC, no template control.

Of the 4 JAK family members (JAK-1/2/3 and TYK2), JAK2 is that most deeply involved in hematopoiesis [1], [2], undergoing physiologic activation by type-1 receptors whose juxtamembrane-cytoplasmic regions it binds by the amino terminal band 4.1, ezrin, radixin, moiesin (FERM) domain mediating cytokine receptor binding (**Fig. 1A**). Without ligand binding, the C-terminal kinase (JH1) of JAK2 is inhibited by the catalytically inactive pseudokinase (JH2) moiety, preventing activation. Ligand binding effects conformational changes which abolish inhibition of JH1 by JH2, allowing phosphorylation and dimerization. A phosphotyrosine domain present in type-1 receptors cooperates with phosphorylated and dimerized JAK2 to enable binding to the SH2 domain of signal transducers and activators of transcription (STAT) family members which themselves undergo phosphorylation, oligomerization, nuclear migration and target gene transcription [3]. Recently, JAK2 has been shown to operate in the nucleus where it activates chromatin by phosphorylating histone H3Y41 to exclude histone HP1α, thereby activating LMO2 and other oncogenes [4].

Several JAK2 fusions have been reported in leukemia/lymphoma without marked lineage preference: BCR-JAK2 in atypical chronic myeloid leukemia (CML), acute myeloid leukemia (AML) and B-cell acute lymphoblastic leukemia (B-ALL); ETV6-JAK2 in pre-B/T-ALL and CML; NFE2-JAK2 in myelodysplastic syndromes (MDS); PAX5/STRN3-JAK2 in childhood ALL; PCM1-JAK2 in myeloid, B-cell and T-cell neoplasias; RPN1-JAK2 in chronic idiopathic myelofibrosis; SEC31A-JAK2 in classical Hodgkin lymphoma; and SSBP2-JAK2 in pre-B ALL [1]. Partner gene protein moieties are believed to support JAK2 oligomerization and constitutive phosphorylation, leading to autonomous signalling. The more indolent and frequent JAK2V617F point mutations which induce myeloproliferative neoplasms occur within exons 12–15 of the pseudokinase domain of JAK2 (**Fig. 1A**), and require the N-terminal FERM domain for binding homodimeric type-I cytokine receptors [5], unlike JAK2 translocation proteins where it is invariably lost.

PCM1 - the JAK2 fusion partner in t(8;9) – functions at the centrosome where it is essential for cell division. Along with PCM1, several tyrosine kinase gene fusion partners physiologically operate at the centrosome. However, speculation that the resultant hybrid proteins promote neoplasia thereat has been challenged since centrosomal targeting by artificial fusion kinases leaves transforming potential unaffected [6]. PCM1 may instead function tumorigenically by inducing JAK2 oligomerization via its coiled coil domains which in the fusion protein remain largely intact

PCM1-JAK2 (reviewed in Hoeller et al. [1]) may be a negative prognostic indicator but symptoms are variable, perhaps due to the relatively wide breakpoint distribution across the SH2-like and pseudokinase domains of JAK2 in affected cases (**Fig. 1A**), C-terminal breakpoints conferring the worst prognoses. t(8;9) has mainly been reported in myeloproliferative neoplasms often accompanied by eosinophilia, followed by B-ALL, with T-cell neoplasia represented by just a single example hitherto [7]. Altered signalling pathways sired by PCM1-JAK2 remain to be defined. Its sibling, ETV6-JAK2, has been characterized in a transgenic mouse model, where STAT-1/3/5 are variously reported as activation targets [8]–[10].

Oncogenic fusion genes present selective targets for RNAi(nterference) conducted by lentiviral transfer of s(mall)h(airpin)RNA plasmids [11], [12]. Leukemia/lymphoma cell lines bearing recurrent fusion genes are ideal vehicles for using RNAi to identify their signalling targets [13]. Here we describe the molecular characterization of t(8;9)(p22;p24) and its effectors in a phenotypically diverse trio of T-cell lymphoma cell lines established during indolent and aggressive disease phases of a cutaneous lymphoma. To decipher the role of t(8;9) in neoplastic signalling, we subjected these cell lines to PCM1-JAK2 knockdown by RNAi, revealing SOCS2 and SOCS3 as key signalling effectors, hitherto mainly regarded JAK signalling inhibitors.

## Materials and Methods

### Cells and patients

Authenticated leukemia/lymphoma cell lines established from patients with T-ALL (ALL-SIL, CCRF-CEM, CML-T1, HPB-ALL, JURKAT, LOUCY, MAT, MOLT-4, MOLT-14, MOLT-16, P12/ICHIKAWA, PEER, RPMI-8402, SUP-T1), ALK-positive anaplastic large cell lymphoma (ALCL) (L-82, SR-786); ALK-negative ALCL (FE-PD), AML (EOL-1, NB-4) and CTCL (HH, together with MY-LA and SE-AX - kindly donated by Dr. Keld Kaltoft, University of Copenhagen, Denmark) - are detailed in Drexler [14] and drawn from the DSMZ public and research repositories. MAC-1/2A/2B were provided by an author (MEK). Clinical particulars of the MAC-donor patient are given by Davis et al. [15] and summarized in **Fig. 1B**. Briefly, a 31-year-old male presented in 1971 with lymphomatoid papulosis (LyP) which by 1983 had progressed to Hodgkin lymphoma when t(8;9) cells were first observed in both dermatopathic lymph node and clinically benign bone marrow. In 1985 an indolent cutaneous lymphoma accompanied by t(8;9)-positive Sézary-like cells was diagnosed, followed in 1987 by aggressive, terminal CD30+ ALCL (ALK-negative). Cell line MAC-1 was established in 1985 during the indolent phase, while MAC-2A and MAC-2B were concurrently established in 1987 from separate cutaneous tumor nodules with anaplastic morphology during advanced disease [15], [16]. Although clonally related, MAC-1/2A/2B are genomically and transcriptionally distinct (Ehrentraut et al., in preparation), supporting the search for signalling targets amid residual commonalities.

We investigated the frequency of JAK2 rearrangements in 217 samples from 200 patients at the Mayo Clinic (Rochester, MN) diagnosed with peripheral T-cell lymphoma (PTCL). The analysis of PTCL tissue specimens was conducted under a protocol approved by the Mayo Clinic Institutional Review Board (IRB). The IRB approved waiver of specific informed consent for this study.

To verify PCM1-JAK2 target gene expression ascertained in T-cells in another setting, bone marrow and peripheral blood from a previously reported t(8;9) patient with a myeloid neoplasm, chronic eosinophilic leukemia (CEL) [17], were also analyzed.

### Cell proliferation assays

Cell lines were prepared for standardized MTT (3-(4,5-dimethylthiazol-2-yl)-2,5-diphenyltetrazolium bromide assays obtained from Sigma (Taufkirchen/Germany). Microcultures of 100 µl were pulsed with 10 µl MTT solution (5 mg/ml in PBS). After 4 h reactions were stopped by adding 120 µl of DMSO/isopropanol. Absorbance was determined at 570 nm by an ELISA reader (Thermo Electron, Vantaa/Finland).

For inhibition of JAK2, cells were treated with TG101348 - a highly selective JAK2 inhibitor [18] - purchased from Active Biochemicals (Hong Kong/PRC). Cells were also treated with pimozide (1-[1-[4,4-bis(4-fluorophenyl)butyl]-4-piperidinyl]-1,3-dihydro-2H-benzimidazole-2-one) (Sigma), a selective inhibitor of STAT5 tyrosine phosphorylation [19]. Experiments were also performed with TGFβ1 (R&D-Systems, Wiesbaden-Nordenstadt/Germany), suberoylanilide hydroxamic acid (SAHA) a histone-deacetylase inhibitor (Sigma) and N-[N-(3,5-Difluorophenacetyl)-L-alanyl]-S-phenylglycine t-butyl ester (DAPT) a γ-secretase inhibitor (Sigma). Substances were dissolved in DMSO diluted to 0.1% in experiments which were performed twice or more in triplicate.

### Genomic characterization

Cytogenetic analyses were conducted as described previously [20]. Imaging was performed using a Zeiss Axioplan (Oberkochen/Germany) microscope configured to a Spectral Karyotyping (SKY) system (ASI Ltd, Migdal Haemek/Israel). Bacterial artificial chromosome (BAC) and fosmid clones were obtained from BACPAC Resources, (Children's Hospital, Oakland/CA). Clinical JAK2 FISH analysis was performed on paraffin sections of PTCLs. Mate pair next generation sequencing (Illumina HiSeq, San Diego/CA) was performed on genomic DNA from MAC-1 and MAC-2A and sequence data mapped to the hg19 reference genome using our published binary indexing algorithm. Mate pairs mapping to 8p22/9p24 were identified, PCR primers designed, and amplicons together with JAK2 exons 12 and 14 Sanger-sequenced.

### Reverse transcriptase (RT-) and quantitative real-time (RQ-) PCR

Total RNA from cell lines was extracted using TRIzol reagent (Invitrogen, Karlsruhe/Germany). cDNA was subsequently synthesized from 5 µg RNA by random priming, using Superscript II (Invitrogen). RQ-PCR was performed by the 7500 Fast Real-time System, using commercial buffer and primer sets (Applied Biosystems, Darmstadt/Germany) or oligonucleotides (MWG Eurofins (Martinsried/Germany). For normalization of expression levels we used TATA box binding protein (TBP). Quantitative analyses were performed in triplicate and repeated twice. Preparation of genomic DNA was performed using the High Pure PCR Template Preparation Kit (Roche Diagnostics, Mannheim/Germany). Genomic PCR was performed using taqpol (Qiagen, Hilden/Germany) and thermocycler TGradient (Biometra, Göttingen/Germany). Oligonucleotides used for qualitative PCR analyses were obtained from MWG Eurofins and sequences given in **Table 1**.

**Table 1:** Oligonucleotides.

Gene	Acc. No.	Forward (5′-3′)	Reverse (5′-3′)	Application
JAK2	NM_004972	-	TGATGGCTCTGAAAGAAGGC	RQ-PCR
			GGTGCTACAACTTTACAGAG	RT-PCR
			TCAATGCATTCAGGTGGTACCC	RT-PCR
PCM1	NM_006197	AACCCTTAGTGCCTAGAGTC	-	RQ-PCR
JAK2	NM_004972	-	TGATGGCTCTGAAAGAAGGC	genomic PCR
PCM1	NM_001172085	TAACCCTACCACTTTGCTG	-	genomic PCR
SOCS2	NM_003877.3	GCGGGAGCTCGGTCAGACAG	CGAAGATTAGTTGGTCCAGCTG	RQ-PCR
SOCS3	NM_003955.3	GCCTATTACATCTACTCCGGG	CGACAGAGATGCTGAAGAGTG	RQ-PCR
PLAGL1	NM_002656	TCCCTGCCTCGCAGCAGATG	CACACTCCTCACTCCCAAAGGC	RQ-PCR
DUSP2	NM_004418	ACTCCAGGGCTCCTGTCTAC	CGGCTGTGATGCCACAGGCC	RQ-PCR
CD3G	NM_000073	GGAAGGGCCTGGCTGTCCTC	TCACAAGTCAGAAGTACCGAACC	RQ-PCR
TIAM1	NM_003253	GCCCTGTGGCTGTTCATCCG	TTGACTTTCTGCGTTTCCCATGG	RQ-PCR
TBP	NM_001172085	CGGAGAGTTCTGGGATTGT	CACGAAGTGCAATGGTCTTT	RQ-PCR

### Construction of PCM1-JAK2 shRNA expression cassettes

A DNA oligonucleotide directed against the fusion region of the human PCM1-JAK2 gene sequence expressed in MAC-cell lines was subjected to BLAST-homology search and thereafter chemically synthesized including 5′ BglII- and a 3′ SalI-restriction site cloning overhangs (BioSpring, Frankfurt/Germany). Sequences are given in **Table 1**. The sense oligonucleotide harbors a poly-T stretch as pol III transcription termination signal. Oligonucleotides were annealed and inserted 3′ of the H1-RNA promoter into the BglII/SalI-digested pBlueScript-derived pH1-plasmid to generate pH1-PCM1-JAK2-1 as described previously [11], [12]. Isolated clones were verified by DNA sequencing. The plasmid pH1-GL4 (control) has been described previously [11], [12]. Construction of lentiviral vectors and preparation of recombinant lentiviral supernatants and lentiviral transduction are detailed in the Supplementary Methods.

### Global gene expression arrays

To parse gene expression, we generated a cDNA expression array using the Affymetrix HGU133plus2 Chip (Santa Clara/CA). These data were supplemented with public array data (https://cabig.nci.nih.gov/community/caArray_GSKdata/). For statistical analyses of primary expression data (CEL-files) and subsequent generation of heat maps we used the R-based Bioconductor Linear Models for Microarray Data (LIMMA) package [21]. For gene annotation we used the Bioconductor homepage (http://www.bioconductor.org). Minimum Information About a Microarray Experiment (MIAME) information for all microarray experiments is available upon request. Gene ontology and pathway analyses were performed using the Broad Institute Gene Set Enrichment Analysis (GSEA) platform (http://www.broadinstitute.org/gsea/index.jsp)./).

### Protein analysis

Western blot analysis was performed by the semi-dry method. Proteins obtained from cell lysates were transferred onto nitrocellulose membranes (Bio-Rad, München/Germany) and blocked with 5% bovine serum albumin (BSA) or milk powder dissolved in phosphate-buffered-saline buffer (PBS) with GAPDH served as loading control [22].

## Results

### t(8;9)(p22;p24) in MAC-1/2A/2B rearranges PCM1 and JAK2

With G-banding (**Fig. 1C**) and SKY (**Fig. 1D**) karyotypes of the three cell lines were resolved to:

**MAC-1:** 45,XY,dup(1)(p31p32),der(4)t(4;5)(p15;p14),t(8;9)(p22;p24),der(11)t(5;11)(p14;p13), der(13)t(8;13)(q11;q32),-18;

**MAC-2A:** 45,X,-Y,del(1)(p34p35),del(5)(q13q15),del(6)(q15),t(8;9)(p22;p24),-10,del(12)(q11q14), der(14)t(14;14)(p11;q13), der(15)t(2;15)(p11;p11),del(16)(q12q21),del(22)(q11q13); and

**MAC-2B:** 45,X,-Y,del(1)(p31p32),der(3)t(3;3)(p25;q11),del(6)(q15),t(8;9)(p22;p24), der(10)t(6;10)(q11;p11),del(12)(q11q14), der(15)t(2;15)(p11;p11).

MAC-1/2A/2B displayed distinct karyotypes where MAC-2A/2B, established concurrently in 1987 from different skin lesions, shared more alterations than MAC-1 established in 1985. Only t(8;9) was conserved throughout, while del(6), del(12) and der(15) were unique to MAC-2A/2B. Interestingly, the t(8;9) had been noted in 1983 in a dermatopathic lymph node and clinically benign bone marrow samples together with various rearrangements affecting chromosome 6 (**Fig. 1B**). FISH analysis of t(8;9) placed the respective breaks at 8p22 and 9p24 inside PCM1 and JAK2, respectively (**Fig. 1E**). Genomic PCR refined this result, pinpointing breaks within intron 38 of PCM1 and intron 16 of JAK2 (**Fig. 1F**). No PCM1 or JAK2 rearrangements were identified in any other ALCL or CTCL cell line analysed.

Interphase FISH excluded JAK2 translocations in 217 PTCLs from 200 patients with: angioimmunoblastic TCL: 60; ALCL, 53 (15 ALK-positive, 23 ALK-negative, and 15 cutaneous); PTCL-not otherwise specified, 59; extranodal NK/TCL, 11; mycosis fungoides, 5; and other cytotoxic PTCLs. Three tumors from 2 patients (ALK-negative, ALCL and PTCL-NOS) exhibited JAK2 amplifications (>10 copies/cell).

### MAC-cell lines carry PCM1-JAK2 fusion

**Figure 2:**
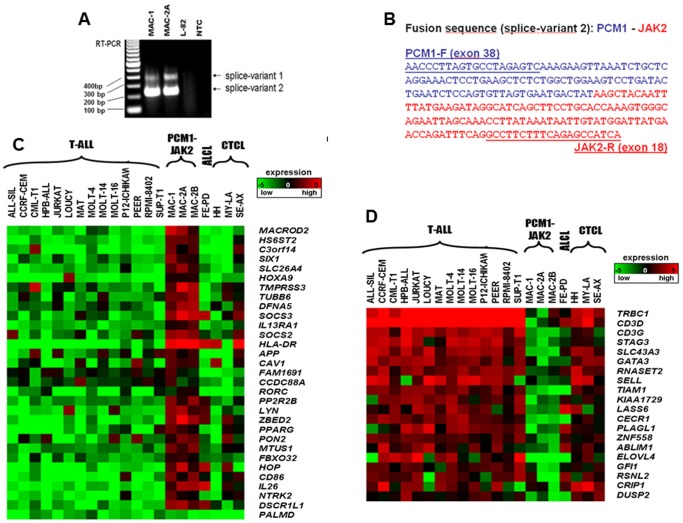
Analysis of gene expression. **A**: Shows RT-PCR analysis with minor (1) and major (2) splice variants which were unique to MAC-1/2A cells; similar data for MAC-2B are not shown. Abbreviation: NTC, no template control. **B**: Fusion sequence of major splice variant and primers used. **C**: Heatmap of top 30 genes differentially expressed in t(8;9) cells, together with a weakly expressed gene PALMD used as negative control in knockdown experiments. Top upregulated genes are listed separately in **Table S3A**. **D**: Heatmap of bottom 20 genes differentially expressed in MAC-1/2A/2B. Top downregulated genes are listed separately in **Table S3B**. To measure the expression profile of the MAC cell lines, we compared the clustered expression of MAC-1/2A/2B, MAC-2A as one group and calculated differential expression versus non-t(8;9) CTCL and T-ALL cell lines. Salient genes combined maximized differential expression and minimized statistical variance. For creation of heat maps we used MeV-Multi-experiment viewer (http://www.tm4.org/mev/node/33).

RT-PCR revealed two chimeric PCM1-JAK2 splice variants in MAC-1/2A (**Fig. 2A**) and MAC-2B (not shown), the major, targeted for knockdown experiments, joined amino acids 1–1947 (of 2024) comprising exons 1–38 (of 39) of PCM1 to amino acids 761–1132 comprising exons 18–25 of JAK2 (**Fig. 2A 12/B**). Both breakpoints lay the farthest downstream hitherto reported (**Fig. 1A**). The predicted fusion protein comprises N-terminal coiled coil domains of PCM1 (amino acids 1 to 1947) joined to the C-terminal tyrosine kinase JH1 and part of the adjoining JH2 pseudokinase domains of JAK2 (amino acids 762 to 1132) - the smallest JAK2 moiety reported to date (**Fig. S1**). Sequencing excluded mutations in exons 12 to 14 (upstream of the breakpoint in MAC-1/2A/2B).

### Expression signatures of MAC-cells unite JAK/STAT with T-effector signalling and disease

Analysis of genes conspicuously (≥10 fold) upregulated in MAC-1/2A/2B compared to 14 control T-ALL cell lines (see Materials and Methods) using the GSEA package highlighted the following ontology categories (detailled in **Table S1A**): “CD133+ hematopoietic stem cells” (p = 0e^0^), “T-cell differentiation” (p = 0e^0^), “recent thymic emigrants” (p≤3.7e^12^). Comparison with CTCL cell lines (**Table S1B**) highlighted: “allograft rejection” (p≤1.5.e^10^); “graft versus host disease” (p≤3.3.1e^10^); “type I diabetes mellitus” (p≤4.6e^10^); “autoimmune thyroid disease” (p≤4.6e^10^); “CD3 and TCR phosphorylation” (p≤5.6e^8^); “PD-1 signalling” (p≤5.0e^7^); “downstream TCR signalling” (p≤1.3e^6^); “co-stimulatory T-cell activation” (p≤1.3e^6^); and “STAT signalling” (p≤7.0e^6^). Downregulated genes highlighted compared to T-ALL (**Table S2A**) included: “autoimmune thyroid disease”, “allograft rejection”, “graft versus host disease”, and “type I diabetes mellitus” all at (p≤0e^0^), together with “JAK-STAT signalling” (p≤3.3e^16^); and “upregulated by IL-2 in CTCL” (p≤3.0e^15^). Comparison with CTCL cell lines (**Table S2B**) highlighted: “T-cell differentiation” (p≤4.0e^7^); “CTL cells mounting immune response” (p≤2.2e^6^); and “immunoregulatory interactions between lymphoid and non-lymphoid cells” (p≤6.6e^6^). Hence overall, conspicuously expressed genes in MAC-1/2A/2B signified JAK/STAT signalling, normal T-cell development, and autoimmune disease.

### PCM1-JAK2 knockdown targets SOCS2 and SOCS3

**Figure 3:**
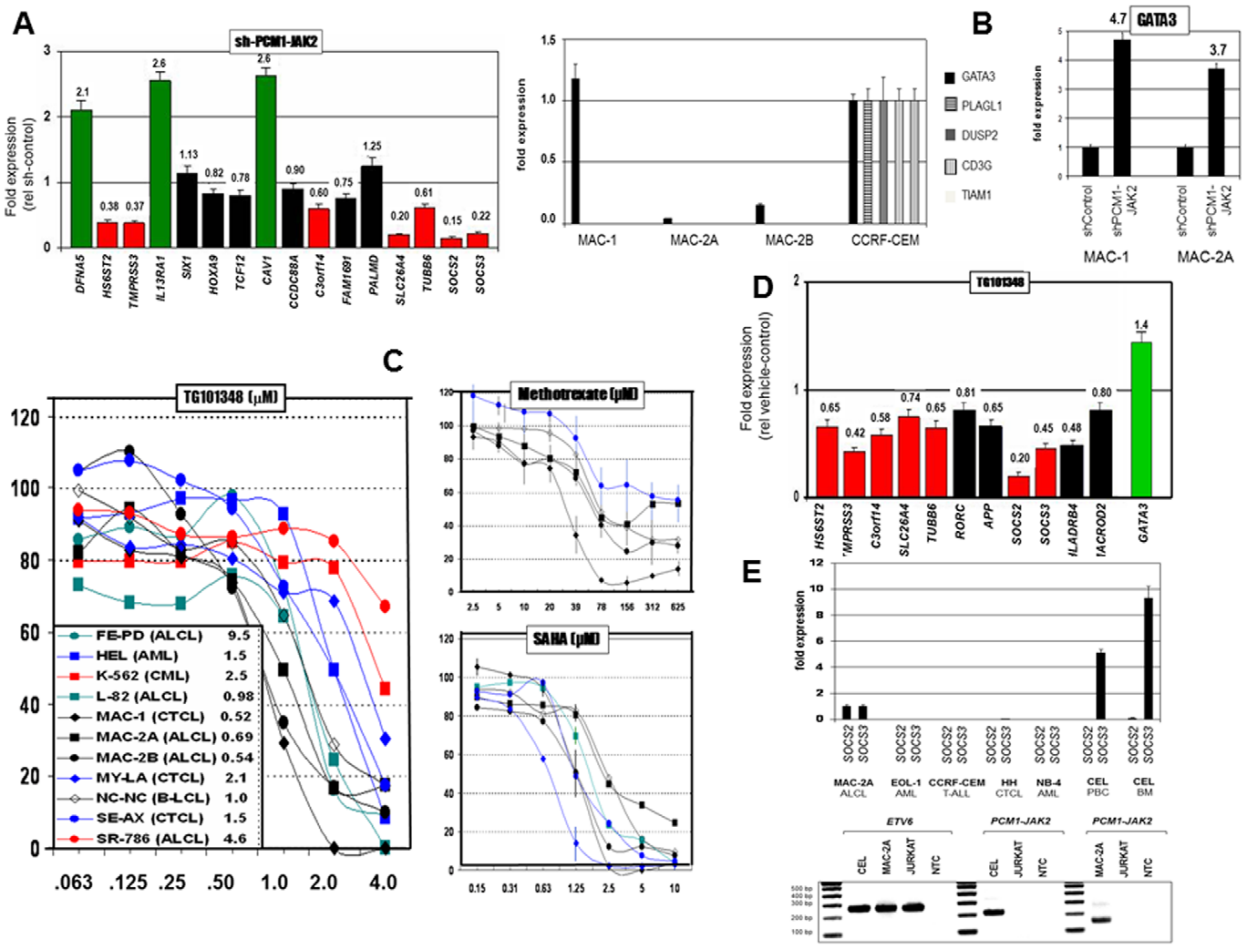
Targets of PCM1-JAK2 signalling. **A**: Shows inhibition (RQ-PCR) of the 15/20 top differentially upregulated genes by PCM1-JAK2 knockdown of MAC-2A cells relative to cells transfected with a control vector. Significantly downregulated genes are shown red, upregulated green, and unaffected black. PALMD which was moderately expressed on the array (**Fig. 2C**) served as a negative control. Data show means of three determinations. Note peak inhibition of SOCS2 followed by SLC26A4 and SOCS3. **B**: Expression of genes conspicuously downregulated in t(8;9) cell lines was validated by RQ-PCR. Contrast inconspicuous expression of GATA3 in indolent phase MAC-1 cells with silencing in aggressive phase MAC-2A/2B cells (left figure). Right figure shows elevated expression of GATA3 in MAC-1 (4.7×) and MAC-2A (3.7×) after PCM1-JAK2 knockdown, indicating negative regulation by PCM1-JAK2. **C**: Shows proliferation responses of t(8;9) and control cell lines to treatments with a selective JAK2 inhibitor (TG101348), methotrexate (amethopterin), and SAHA (vorinostat). Note lowest IC50 values (inset) of MAC-1/2A/2B to TG101348, while drugs currently used in therapy flatline (methotrexate) or yield nondescript results (SAHA). Data show means of two or more experiments performed in triplicate with standard errors (omitted from TG101348 for clarity). **D**: Shows pharmacological inhibition (RQ-PCR) by TG101348 (1 µM 72 h) of top 7 PCM1-JAK2 knockdown targets (red) and 4 additional genes which yielded inconsistent results in knockdown experiments (black). Note peak inhibition of SOCS2 and SOCS3 confirming knockdown data. Figure also shows upregulation of GATA3 (green) consistent with sh-RNA data. **E**: Relative SOCS2/SOCS3 expression (RQ-PCR) in a chronic eosinophilic leukemia (CEL) patient with PCM1-JAK2, with expression in MAC-2A set to unity. Upper figure shows massive upregulation of SOCS3 (but not SOCS2) in patient peripheral blood lymphocytes (PBC) and bone marrow (BM), while control AML (including EOL-1 established from a patient with an acute eosiniphilic leukemia lacking PCM1-JAK2 rearrangement), CTCL, and T-ALL cells expressed neither gene. Lower figure shows confirmation of PCM1-JAK2 expression in CEL patient PBC by RT-PCR. ETV6 served as control.

To identify specific PCM1-JAK2 targets, the most conspicuously expressed genes revealed by analysis of the array data were shortlisted. Due to their lower variances, conspicuous upregulation (**Fig. 2C**) was more easily discernible than downregulation (**Fig. 2D**). For a full list of conspicuously upregulated and downregulated genes in MAC-1/2A/2B see **Tables S3A/B**. As well as putative PCM1-JAK2 targets, shortlisted genes probably included those activated developmentally and pathologically by other means in CTCL cells, e.g. CD86 and HLA-DR loci, associated with antigen presenting cells and autoimmune disease, respectively. To identify bona fide translocation targets, responses of shortlisted candidates to PCM1-JAK2 knockdown by siRNA were validated by RQ-PCR in MAC-2A cells (**Fig. 3A**). Of 16 genes analyzed, 7 demonstrated significant inhibition by RNAi (**Table 2**). SOCS2 and SOCS3, respectively downregulated to 15% and 22% of control levels, are integral to JAK-STAT signalling, while the next SLC26A4 (20% expression) controls ion-transport. Three genes (CAV1, DFNA5, IL13RA1) were consistently upregulated in knockdown experiments, implying that PCM1-JAK2 in these cases was acting in an inhibitory capacity.

**Table 2:** Top PCM1-JAK2 transcriptional target genes.

	Gene	Name	Modulation		TFBS	Annotation
			RNAi	TG-101348		
1	SOCS2	Suppressor of cytokine signaling 2	0.15	0.2	AHR STAT1 STAT1alpha STAT1β STAT5A STAT5B PPAR-gamma1 PPAR-γ2 FOXC1 RORα2	-Suppresses JAK signalling by direct protein interaction-Contains KIR domain-See text for details
2	SOCS3	Suppressor of cytokine signaling 3	0.22	0.45	C/EBPβ GR-β GR-α RFX1 PPAR-γ1 PPAR-γ2 STAT3 STAT5 c-Myb HOXA9B MEIS1	-Suppresses JAK signalling by direct protein interaction-Contains no KIR domain-See text for details
3	TMPRSS3	transmembrane protease, serine 3	0.37	0.42	ECR1 C/EBPβ NF-κB1 NRSF form 1 NRSF form 2 p53 STAT3 BACH1 c-Myc MAX1	-Ubiquitously expressed transmembrane serum protease which regulates iron homeostasis-Overexpression defines a cytotoxic T-lymphocyte subset.-Upregulated by JAK-STAT signalling activated by IL-29 in melanoma-Maps to diabetes risk locus at chr. 21q21
4	SLC26A4	solute carrier family 26, member 4	0.20	0.74	MEIS1 HOXA9B STAT5B STAT5A NKX3-1 FRA1	-DNA binding protein (predicted)-Encodes anion transporter pendrin expressed in thyroid and lung where it is pro-inflammatory-Upregulated in ALCL (www.ebi.ac.uk/)
5	HS6ST2	heparan sulfate 6-O-sulfotransferase 2	0.38	0.65	c-MYC MAX1 MYOD HNF-1A ATF-2 RORα2 ARNT TAL1 E47 c-MYB	-Normally expressed in prostate and brain-Catalyses transfer of sulfate to heparan sulfate-Regulates development, angiogenesis, blood coagulation and metastasis
6	C3ORF14	chromosome 3 open reading frame 14	0.60	0.58	STAT1β STAT1 STAT1α FOXD1 STAT3 STAT2 STAT5A STAT5B STAT6 STAT4	-Maps near FHIT/FRA3B at chr. 3p14-Conspicuously expressed in multiple myeloma cells responding to knockdown of MMSET/WHSC1 by siRNA
7	TUBB6	tubulin, beta 6	0.61	0.64	BACH1 AP-1 c-FOS c-JUN FRA1 JUNB JUND p300 CUTL1 BACH2	- Conspicuously upregulated in chronic lymphocytic leukemia with t(14;19)(q32;q13) and unmutated IgVH. Several anticancer drugs inhibit beta-tubulins blocking cell division
8	GATA3	GATA binding protein 3	3.7	1.4	GATA3, AML1A, GATA6, IK2, CMYB, LMO2, PAX5, ZIC3, HTF,	- Contains two zinc fingers and is a key regulator of T-cell development.- See text for details

Shows top validated targets ranked by combining transcriptional modulation (Rq-PCR) after PCM1-JAK2 knockdown in MAC-2A cells with that due to JAK2 inhibition with TG101348. Modulation data show expression of target genes in MAC-2A cells (ranked by combined responses) after knockdown or subtoxic TG101348 treatment (1 µM 72h) compared to controls.

### GATA3 is silenced by PCM1-JAK2

Further evidence that PCM1-JAK2 effects gene silencing is provided by GATA3, well expressed in indolent phase MAC-1 cells, but downregulated in aggressive phase MAC-2A/2B (**Fig. 3B**). Following PCM1-JAK2 knockdown, GATA3 expression was increased in both MAC-1 (4.7×) and MAC-2A (3.7×) showing that the fusion gene occupies an inhibitory role in GATA3 regulation (**Fig. 3B**).

### PCM1-JAK2 cells are maximally sensitive to selective JAK2 inhibition

Of 11 cell lines tested, proliferation of MAC-1/2A/2B cells was the most sensitive to the selective JAK2 inhibitor TG101348 (**Fig. 3C**) with IC50 values below those lacking JAK2 rearrangement. The steep dose-responses elicited by TG101348 contrasted with both methotrexate and SAHA (**Fig. 3C**) clinically applied in CTCL where cell lines either flatlined above 156 µM, or overlapped non-t(8;9) examples.

### Selective JAK2 inhibition mimics PCM1-JAK2 knockdown

To corroborate knockdown data, expression of 7 inhibited genes, together with 4 initially untested in RNAi cells, was measured in t(8;9) MAC-2A cells after TG101348 treatment. Results supported RNAi data (**Fig. 3D** **,** **Table 2**). SOCS2 – the leading PCM1-JAK2 target – also proved the most sensitive to pharmacological inhibition with expression reduced to 20% control levels, while that of SOCS3 was reduced to 45%. Inhibition of SLC36A4 reached only 0.74. Expression of GATA3 (green), silenced by PCM1-JAK2 in MAC2A/B, was partially restored by TG101348 treatment, consistent with the sh-PCM1-JAK2 data consolidating its role as a translocation target.

Collectively, these findings show that expression of SOCS2 followed by SOCS3 requires PCM1-JAK2. SOCS2 is a negative regulator of cytokine signalling, inducible by cytokines, including erythropoietin, GM-CSF, IL10 and interferon (IFN)-gamma. Both SOCS2 (2 and 13 sites) and SOCS3 (10 and 9 sites) carry TFBS for STAT3 and STAT5A, respectively (http://www.sabiosciences.com/chipqpcrsearch.php?app=TFBS; **Table 2**, **Fig. S2**) and therefore both plausible JAK/STAT signalling targets. Given their known roles in JAK-STAT signalling, SOCS2 and SOCS3 were deemed the leading candidates among those listed in **Table 2** and are discussed in detail in the next section.

Remaining candidates have yet to be implicated as upstream signalling effectors but may operate downstream. These were in order of combined knockdown/JAK2 inhibition: transmembrane protease, serine 3 (TMPRSS3) is a ubiquitously expressed transmembrane serum protease which regulates iron homeostasis, located at or near a type I diabetes susceptibility locus at chromosome 21q21; solute carrier family 26, member 4 (SLC26A4) which encodes the anion transporter pendrin which is upregulated in ALCL patients at diagnosis (www.ebi.ac.uk/); chromosome 3 open reading frame 14 (C3ORF14) (alias LOC57415, FLJ94553, FLJ17473) - a predicted DNA binding protein; tubulin, beta 6 (TUBB6) normally expressed in neurons; and heparan sulfate 6-O-sulfotransferase 2 (HS6ST2) which catalyzes the transfer of sulfate from adenosine 3′-phosphate, 5′-phosphosulfate to heparan sulphate when regulating developmental processes, angiogenesis, blood coagulation and tumor metastasis. Some of these candidates carry STAT-3/5 TFBS, albeit of untested functionality.

In addition to PCM1-JAK2 shown above (**Fig. 3A/D**), GATA3 is physiologically regulated by multiple factors, their expression in MAC-1/2A/2B shown in **Fig. S3** together with GATA3. Differential expression of TGFβ1 (negative regulator) followed by NOTCH2 (positive regulator) correlated best with GATA3 (left), which nevertheless, withstood treatment with either TGFβ or DAPT (Notch inhibitor), implying that in aggressive phase MAC-2A/2B cells silencing of GATA3 is otherwise regulated (**Fig. S3**).

### SOCS3 is present at high levels in both lymphoid and myeloid neoplasia with PCM1-JAK2

To verify both leading PCM1-JAK2 targets SOCS2 and SOCS3, we measured their expression in a CEL patient with t(8;9)/PCM1-JAK2 [22]. We found that SOCS3, but not SOCS2, was highly expressed in leukemic blood and bone marrow, while leukemia/lymphoma cell lines lacking PCM1-JAK2, including acute eosinophilic leukemia, expressed neither gene (**Fig. 3E**). Thus, SOCS3 accompanies t(8;9)/PCM1-JAK2 in both myeloid neoplastic cells and T-lymphoid cells, while SOCS2 may be restricted to the latter.

### STAT5 activation requires PCM1-JAK2 signalling

**Figure 4:**
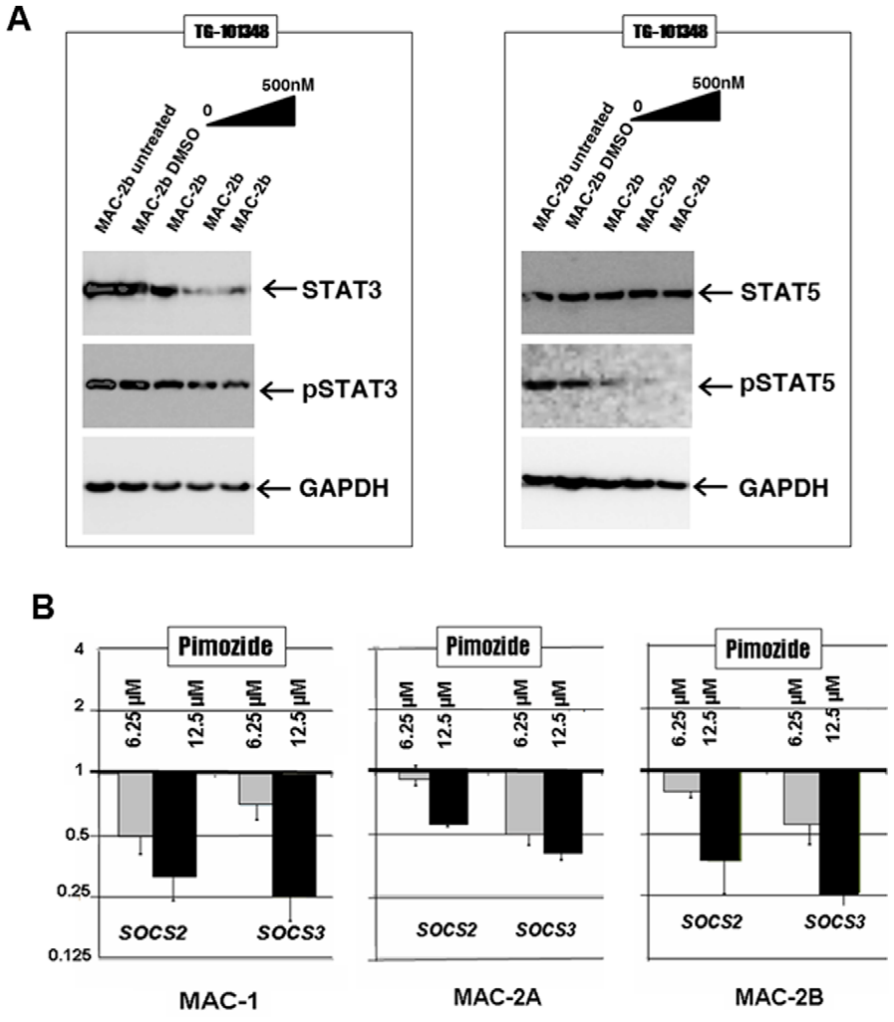
STAT involvement. A: Western blot analysis of STAT3/pSTAT3 and STAT5/pSTAT5 in MAC-2B cells subjected to JAK2 inhibition by exposure to ascending (moderate) concentrations of TG101348 over 72 h. Note dose dependent loss of STAT3 and pSTAT5 only. GAPDH served as loading control. B: Shows expression of SOCS2 and SOCS3 in MAC-1/2A/2B cells exposed to two concentrations of STAT5 inhibitor pimozide for 72 h with vehicle controls set to unity. Note dose dependent losses of SOCS3 expression, followed by SOCS2. Abbreviations: anaplastic large cell lymphoma (ALCL); acute myeloid leukemia (AML); B-lymphoblastoid cell line (B-LCL); cutaneous T-cell lymphoma (CTCL); peripheral T-cell lymphoma (PTCL); T-cell acute lymphoblastic leukemia (T-ALL).

The effects of JAK2 inhibition on JAK-STAT signalling were further investigated by western blotting. **Fig. 4A** depicts protein expression of STAT3 (left) and STAT5 (right). Both STAT5/pSTAT5 and STAT3/pSTAT3 are well expressed and constitutively activated in t(8;9) cells. However, while activation of STAT5 was sensitive to JAK2 inhibition in a dose-dependent manner, STAT3 phosphorylation withstood JAK2 inhibition even though protein, like mRNA (not shown), expression was lowered. These data pinpoint STAT5 activation as a key oncogenic mediator of PCM1-JAK2 signalling followed by STAT3 expression.

### SOCS2 and SOCS3 expression requires activated STAT5

Inhibition of STAT5 activation by treatment with the STAT5 inhibitor pimozide was accompanied by dose-dependent losses of SOCS2/3 expression in all three PCM1-JAK2 cell lines, MAC-1/2A/2B (**Fig. 4B**), confirming both genes as STAT5 activation targets. Downregulation of SOCS2/SOCS3 by siRNA was unattended by inhibition of JAK2 or PCM1-JAK2 (not shown), discounting roles in regulating wild type JAK2.

## Discussion

JAK2-translocation neoplasms are of significant biologic and therapeutic interest, notably since their neoplastic signalling depends on a defined, druggable target,and is distinguished from the better understood JAK2617F-mutation disease by their greater clinical and protein-structural heterogeneity,. MAC-1/2A/2B are the first cell lines shown to carry a JAK2 translocation, and the extreme C-terminal placement of the breakpoint within JAK2 clusters with those previously reported in T-cell neoplasms. Gene ontology profiling confirms their fitness to model T-cell neoplasias with a conspicuous inflammatory component as reported in CTCL. MAC-1/2A/2B by simultaneously modelling CTCL and JAK2-translocation neoplasia should be useful tools for preclinical development and assessment of novel targeted therapies.

In our panel of hematopoietic neoplasias, MAC-1/2A/2B were conspicuously sensitive to a selective JAK2 inhibitor TG101348, surpassing even JAK2V617F mutation cells, while responding unremarkably to methotrexate or SAHA used clinically in treating CTCL. Although more than 20 clinical trials are underway, JAK2 inhibitors have only been approved for use in the USA since November 2011 [23]. The effectiveness of current – palliative - JAK2 inhibitors is limited by their equi-potency against normal and mutant JAK2V617F protein [1]. Very recently, a CEL patient with PCM1-JAK2 was successfully treated with a JAK1/2 inhibitor ruxolitinib [24], a drug which hitherto showed modest activity against myelofibrosis with JAK2V617F [25]. And while TG101348 has also proved palliative against JAK2V617F-induced myelofibrosis [18], its efficacy against JAK2-translocation leukemia/lymphoma remains untested. We have already described JAK2V617F cell line models [26], and their moderate responses to TG101348 when compared to PCM1-JAK2 examples also supports evaluation of selective JAK2 inhibitors in the latter disease context.

Of all JAK2 translocations, ETV6-JAK2 has been studied in most detail, thanks to mouse and zebrafish models. In pre-B and T-lymphoid forms ETV6 is respectively fused with JAK2 exons 17 and 19, both retaining the JH1 tyrosine kinase domain, while in the single atypical CML case reported the breakpoint lay farther upstream to include the JH2 pseudokinase domain [27], [28]. Our combined shRNA and selective pharmacological inhibitory data highlight the JAK2 kinase domain as the active moiety, while the extreme C-terminal placement of the JAK2 breakpoint redefines the minimal tyrosine kinase transforming region.

STAT5 activation is required for transformation by ETV6-JAK2. All three lineage-related breakpoint variants cited above induced STAT5 phosphorylation in the BA/F3 mouse cell line model [27]–[29]. In mice transplanted with bone marrow transduced to express ETV6-JAK2, activation of STAT5 is required for myelo-/lympho-proliferative disease to emerge [29]. Our observation of STAT5 phosphorylation in PCM1-JAK2 cells contingent upon JAK2 activity strongly suggests that both translocations operate via STAT5, supporting a paradigm of STAT5 activation in JAK2-neoplasia.

The conclusion that SOCS2 and SOCS3 are both key signalling targets of t(8;9), is supported by both fusion gene knockdown and selective pharmacological inhibition data. Both SOCS loci bear multiple STAT5A TFBS which are reportedly functional [30], suggesting a mechanistic basis for SOCS2/3 upregulation by PCM1-JAK conducted via STAT5. However, while transcriptional analysis of a CEL patient with PCM1-JAK2 confirmed upregulation of SOCS3, SOCS2 expression remained low, highlighting the former as the primary target of PCM1-JAK2. Interestingly, SOCS2-/- mice display marked eosinophilia [31], raising the possibility that low SOCS2 expression in the CEL patient is lineage restricted.

SOCS3 serves as a negative regulator of JAK-STAT signalling: either by direct JAK binding where its kinase inhibitory region (KIR) directly inhibits or decoys the structurally related JAK-tyrosine kinase domain [32], or by blocking phosphotyrosine sites on cytokine receptors to initiate their lysosomal or proteosomal degradation [33], [34]. Nevertheless, upregulation of SOCS3 has been reported in CTCL and other malignancies in association with elevated JAK-STAT signalling [35], and in JAK2V617F positive myeloproliferative disorders together with STAT3 and STAT5B [36], consistent with our findings. Although, SOCS3 has been reported as a feedback target of JAK-STAT signalling via IL9 in a mouse T-cell lymphoma [37], our unpublished findings discount a similar mechanism in MAC-1/2A/2B (Ehrentraut et al., in preparation). In CTCL cells SOCS3 overexpression inhibits apoptosis induced by interferon-α [38]. Thus, SOCS3 is versatile, acting as tumor suppressor, tumor promoter, or immunomodulator according to context.

The actions of SOCS2 like those of SOCS3 are varied. While also a JAK-STAT signalling inhibitor, SOCS2 lacks the KIR domain found in SOCS3, proferring an explanation for their diverse modes of action. Although, Schultheis et al. [39] reported SOCS2 activation in CML, Hansen et al. [40] have shown its redundance for generation of CML in a BCR-ABL1 driven mouse model, implying that feedback regulation by SOCS2 is deficient in this setting. Underlying these discrepancies may be that SOCS2 inhibits STAT5 induced by growth hormone signalling at lower transcription levels, but at high levels stimulates STAT5 [41].

SOCS2 and SOCS3 may act antagonistically: SOCS2 controls T-cell polarization, repressing Th2, but supporting Th17 differentiation, while SOCS3 plays opposing roles [42]. It has been shown that SOCS2 increases and prolongs STAT5 phosphorylation induced by IL-2/3 [41]–[43] which, in turn, increases proliferation, while SOCS3 reduces pSTAT5 levels, JAK2 mutant cases excepted [44]. Our findings exclude JAK2 and PCM1-JAK2 as SOCS2/3 targets in MAC-cells, their actual targets currently under investigation by siRNA screening.

Although, our data show that GATA3 is negatively regulated by PCM1-JAK2 in MAC-cells, GATA3 silencing in MAC-2A/2B seemingly requires additional regulatory input. Physiological GATA3 expression is supported by BHLHB38, IL-4/STAT6, NFKB, NOTCH and TCF1, and inhibited by RUNX1, TBX21 and TGFβ1 [45]. GATA3 promotes functional T-cell plasticity [46] as retained in MAC-1 cells and in another early-phase CTCL cell line expressing GATA3, but lost in MAC-2A/2B where GATA3 is silenced (Ehrentraut et al., in preparation).

While this is the first report of a JAK2 activating rearrangement in CTCL, prominent JAK3 signalling via STAT3 in CTCL cells has been described [47], [48]. Both STAT3 and STAT5 may be expressed in T-helper cells and CTCL, again serving antagonistically in lineage fate determination [48]. Recently, risk susceptibility loci for Crohn's disease and psoriasis have been mapped at or near JAK2 and STAT3 [49]. Suppression of pSTAT3 following topical application of JAK-1/2 inhibitor in cutaneous inflammation has been reported [50], encouraging hopes that selective JAK inhibitors may play a role in treating inflammatory skin disease as well as JAK2 translocation associated neoplasia.

In summary, we investigated JAK2-translocation induced malignancy by shRNA-mediated knockdown to reveal signalling by SOCS2 and SOCS3 via STAT5 as prime downstream effectors of PCM1-JAK2 This study validates the first cell line models for JAK2-translocation malignancy, providing tools for investigating neoplastic signalling of this entity in a human context. Taken together with other reports, our findings suggest that STAT5 signalling may be a general feature of JAK2-translocation leukemia/lymphoma and link clinical aspects of cutaneous lymphoma with physiological T-cell differentiation.

## Supporting Information

Figure S1 Amino acid sequences at breakpoints in PCM1 and JAK2. Gives amino acid sequences of PCM1 and JAK2, those retained in the fusion product are shown in black.
(TIF)

Figure S2 Transcription factor binding sites of STAT3 and STAT5. The figure is drawn from the UCSC browser (HG19) and covers *SOCS2* and *SOCS3*. Note presence of TFBS for STAT3 and STAT5 at both *SOCS* loci.
(TIF)

Figure S3 Differential expression of GATA3 regulators in MAC-1/2A/2B cells. Microarray heatmap (left) shows expression of known GATA3 regulators (arrows showing whether positive/negative). *NOTCH2* (positive) and *TGF*β*1* (negative) regulators like GATA3 are differentially regulated in MAC-1 and MAC-2A/B cells. However, normalized GATA3 expression (RQ-PCR) after 72h treatment with neither DAPT (γ-secretase inhibitor) nor TGFβ1 evidence modulation (right). Vehicle control data (black) shown left of treatment.
(TIF)

Table S1 Ontology profiling of genes upregulated in MAC-1/2A/2B versus control T-ALL (A) or CTCL (B) cell lines listed in the Materials and Methods. Shows ontology profiling using the Gene Set Enrichment (GSEA) engine hosted by the Broad Institute (http://www.broadinstitute.org/gsea/index.jsp).
(XLSX)

Table S2 Ontology profiling of genes downregulated in MAC-1/2A/2B versus control T-ALL (A) or CTCL (B) cell lines listed in the Materials and Methods. Shows ontology profiling using the Gene Set Enrichment (GSEA) engine hosted by the Broad Institute (http://www.broadinstitute.org/gsea/index.jsp).
(XLSX)

Table S3 Top upregulated (A) and downregulated (B) genes in MAC-1/2A/2B cells. Shows 400 genes most conspicuously expressed when compared to control T-ALL and CTCL cell lines listed in the Materials and Methods.
(XLSX)

Methods S1 (DOCX)

## References

[1] Hoeller S, Walz C, Reiter A, Dirnhofer S, Tzankov A (2011) PCM1-JAK2-fusion: a potential treatment target in myelodysplastic-myeloproliferative and other hemato-lymphoid neoplasms. Expert Opin Ther Targets 15: 53–62. 21091042 10.1517/14728222.2011.538683

[2] Chen E, Staudt LM, Green AR (2012) Janus kinase deregulation in leukemia and lymphoma. Immunity 36: 529–541. 22520846 10.1016/j.immuni.2012.03.017 PMC7480953

[3] Radtke S, Haan S, Jorissen A, Hermanns HM, Diefenbach S, et al (2005) The Jak1 SH2 domain does not fulfill a classical SH2 function in Jak/STAT signaling but plays a structural role for receptor interaction and up-regulation of receptor surface expression. J Biol Chem 280: 25760–25768. 15894543 10.1074/jbc.M500822200

[4] Dawson MA, Bannister AJ, Gottgens B, Foster SD, Bartke T, et al (2009) JAK2 phosphorylates histone H3Y41 and excludes HP1alpha from chromatin. Nature 461: 819–822. 19783980 10.1038/nature08448 PMC3785147

[5] Lu X, Levine R, Tong W, Wernig G, Pikman Y, et al (2005) Expression of a homodimeric type I cytokine receptor is required for JAK2V617F-mediated transformation. Proc Natl Acad Sci U S A 102: 18962–18967. 16365288 10.1073/pnas.0509714102 PMC1323216

[6] Bochtler T, Kirsch M, Maier B, Bachmann J, Klingmuller U, et al (2012) Centrosomal targeting of tyrosine kinase activity does not enhance oncogenicity in chronic myeloproliferative disorders. Leukemia 26: 728–735. 22015771 10.1038/leu.2011.283

[7] Adelaide J, Perot C, Gelsi-Boyer V, Pautas C, Murati A, et al (2006) A t(8;9) translocation with PCM1-JAK2 fusion in a patient with T-cell lymphoma. Leukemia 20: 536–537. 16424865 10.1038/sj.leu.2404104

[8] Ho JM, Beattie BK, Squire JA, Frank DA, Barber DL (1999) Fusion of the ets transcription factor TEL to Jak2 results in constitutive Jak-Stat signaling. Blood 93: 4354–4364. 10361134

[9] Lacronique V, Boureux A, Monni R, Dumon S, Mauchauffe M, et al (2000) Transforming properties of chimeric TEL-JAK proteins in Ba/F3 cells. Blood 95: 2076–2083. 10706877

[10] Schwaller J, Frantsve J, Aster J, Williams IR, Tomasson MH, et al (1998) Transformation of hematopoietic cell lines to growth-factor independence and induction of a fatal myelo- and lymphoproliferative disease in mice by retrovirally transduced TEL/JAK2 fusion genes. EMBO J 17: 5321–5333. 9736611 10.1093/emboj/17.18.5321 PMC1170859

[11] Scherr M, Battmer K, Blomer U, Schiedlmeier B, Ganser A, et al (2002) Lentiviral gene transfer into peripheral blood-derived CD34+ NOD/SCID-repopulating cells. Blood 99: 709–712. 11781260 10.1182/blood.v99.2.709

[12] Scherr M, Battmer K, Ganser A, Eder M (2003) Modulation of gene expression by lentiviral-mediated delivery of small interfering RNA. Cell Cycle 2: 251–257. 12734435

[13] MacLeod RA, Nagel S, Scherr M, Schneider B, Dirks WG, et al (2008) Human leukemia and lymphoma cell lines as models and resources. Curr Med Chem 15: 339–359. 18288989 10.2174/092986708783497319

[14] Drexler HG (2000) The Leukemia-Lymphoma Cell Lines FactsBook. San Diego: Academic Press.

[15] Davis TH, Morton CC, Miller-Cassman R, Balk SP, Kadin ME (1992) Hodgkin's disease, lymphomatoid papulosis, and cutaneous T-cell lymphoma derived from a common T-cell clone. N Engl J Med 326: 1115–1122. 1532439 10.1056/NEJM199204233261704

[16] Kadin ME, Cavaille-Coll MW, Gertz R, Massague J, Cheifetz S, et al (1994) Loss of receptors for transforming growth factor beta in human T-cell malignancies. Proc Natl Acad Sci U S A 91: 6002–6006. 8016105 10.1073/pnas.91.13.6002 PMC44125

[17] Prochorec-Sobieszek M, Nasilowska-Adamska B, Borg K, Kopec I, Kos-Zakrzewska K, et al (2012) Chronic eosinophilic leukemia with erythroblastic proliferation and the rare translocation t(8;9)(p22;p24) with PCM1-JAK2 fusion gene: a distinct clinical, pathological and genetic entity with potential treatment target? Leuk Lymphoma 53: 18241827. 10.3109/10428194.2012.661856 22288769

[18] Pardanani A, Gotlib JR, Jamieson C, Cortes JE, Talpaz M, et al (2011) Safety and efficacy of TG101348, a selective JAK2 inhibitor, in myelofibrosis. J Clin Oncol 29: 789–796. 21220608 10.1200/JCO.2010.32.8021 PMC4979099

[19] Nelson EA, Walker SR, Weisberg E, Bar-Natan M, Barrett R, et al (2011) The STAT5 inhibitor pimozide decreases survival of chronic myelogenous leukemia cells resistant to kinase inhibitors. Blood 117: 3421–3429. 21233313 10.1182/blood-2009-11-255232 PMC3069678

[20] MacLeod RA, Kaufmann M, Drexler HG (2007) Cytogenetic harvesting of commonly used tumor cell lines. Nat Protoc 2: 372–382. 17406598 10.1038/nprot.2007.29

[21] Smyth GK (2005) Limma: linear models for microarray data. In: Gentleman RC, V.; Dudoit, S.; Irizarry, R.; Huber, W., editor. Bioinformatics and Computational Biology Solutions using R and Bioconductor. New York: Springer. pp. 397–420.

[22] Quentmeier H, Geffers R, Jost E, Macleod RA, Nagel S, et al (2008) SOCS2: inhibitor of JAK2V617F-mediated signal transduction. Leukemia 22: 2169–2175. 18769447 10.1038/leu.2008.226

[23] Tefferi A (2012) JAK inhibitors for myeloproliferative neoplasms: clarifying facts from myths. Blood 119: 2721–2730. 22279053 10.1182/blood-2011-11-395228

[24] Lierman E, Selleslag D, Smits S, Billiet J, Vandenberghe P (2012) Ruxolitinib inhibits transforming JAK2 fusion proteins in vitro and induces complete cytogenetic remission in t(8;9)(p22;p24)/PCM1-JAK2-positive chronic eosinophilic leukemia. Blood 120: 1529–1531. 22899477 10.1182/blood-2012-06-433821

[25] Harrison C, Verstovsek S, McMullin MF, Mesa R (2012) Janus kinase inhibition and its effect upon the therapeutic landscape for myelofibrosis: from palliation to cure? Br J Haematol 157: 426–437. 22463737 10.1111/j.1365-2141.2012.09108.x PMC8721529

[26] Quentmeier H, MacLeod RA, Zaborski M, Drexler HG (2006) JAK2 V617F tyrosine kinase mutation in cell lines derived from myeloproliferative disorders. Leukemia 20: 471–476. 16408098 10.1038/sj.leu.2404081

[27] Lacronique V, Boureux A, Valle VD, Poirel H, Quang CT, et al (1997) A TEL-JAK2 fusion protein with constitutive kinase activity in human leukemia. Science 278: 1309–1312. 9360930 10.1126/science.278.5341.1309

[28] Peeters P, Raynaud SD, Cools J, Wlodarska I, Grosgeorge J, et al (1997) Fusion of TEL, the ETS-variant gene 6 (ETV6), to the receptor-associated kinase JAK2 as a result of t(9;12) in a lymphoid and t(9;15;12) in a myeloid leukemia. Blood 90: 2535–2540. 9326218

[29] Schwaller J, Parganas E, Wang D, Cain D, Aster JC, et al (2000) Stat5 is essential for the myelo- and lymphoproliferative disease induced by TEL/JAK2. Mol Cell 6: 693–704. 11030348 10.1016/s1097-2765(00)00067-8

[30] Vidal OM, Merino R, Rico-Bautista E, Fernandez-Perez L, Chia DJ, et al (2007) In vivo transcript profiling and phylogenetic analysis identifies suppressor of cytokine signaling 2 as a direct signal transducer and activator of transcription 5b target in liver. Mol Endocrinol 21: 293–311. 17008382 10.1210/me.2006-0096

[31] Knosp CA, Carroll HP, Elliott J, Saunders SP, Nel HJ, et al (2011) SOCS2 regulates T helper type 2 differentiation and the generation of type 2 allergic responses. J Exp Med 208: 1523–1531. 21646394 10.1084/jem.20101167 PMC3135359

[32] Babon JJ, Kershaw NJ, Murphy JM, Varghese LN, Laktyushin A, et al (2012) Suppression of cytokine signaling by SOCS3: characterization of the mode of inhibition and the basis of its specificity. Immunity 36: 239–250. 22342841 10.1016/j.immuni.2011.12.015 PMC3299805

[33] Croker BA, Kiu H, Nicholson SE (2008) SOCS regulation of the JAK/STAT signalling pathway. Semin Cell Dev Biol 19: 414–422. 18708154 10.1016/j.semcdb.2008.07.010 PMC2597703

[34] Piessevaux J, Lavens D, Peelman F, Tavernier J (2008) The many faces of the SOCS box. Cytokine Growth Factor Rev 19: 371–381. 18948053 10.1016/j.cytogfr.2008.08.006

[35] Brender C, Nielsen M, Kaltoft K, Mikkelsen G, Zhang Q, et al (2001) STAT3-mediated constitutive expression of SOCS-3 in cutaneous T-cell lymphoma. Blood 97: 1056–1062. 11159537 10.1182/blood.v97.4.1056

[36] Irino T, Uemura M, Yamane H, Umemura S, Utsumi T, et al (2011) JAK2 V617F-dependent upregulation of PU.1 expression in the peripheral blood of myeloproliferative neoplasm patients. PLoS One 6: e22148. 21789226 10.1371/journal.pone.0022148 PMC3138766

[37] Lejeune D, Demoulin JB, Renauld JC (2001) Interleukin 9 induces expression of three cytokine signal inhibitors: cytokine-inducible SH2-containing protein, suppressor of cytokine signalling (SOCS)-2 and SOCS-3, but only SOCS-3 overexpression suppresses interleukin 9 signalling. Biochem J 353: 109–116. 11115404 PMC1221548

[38] Brender C, Lovato P, Sommer VH, Woetmann A, Mathiesen AM, et al (2005) Constitutive SOCS-3 expression protects T-cell lymphoma against growth inhibition by IFNalpha. Leukemia 19: 209–213. 15618960 10.1038/sj.leu.2403610

[39] Schultheis B, Carapeti-Marootian M, Hochhaus A, Weisser A, Goldman JM, et al (2002) Overexpression of SOCS-2 in advanced stages of chronic myeloid leukemia: possible inadequacy of a negative feedback mechanism. Blood 99: 1766–1775. 11861294 10.1182/blood.v99.5.1766

[40] Hansen N, Agerstam H, Wahlestedt M, Landberg N, Askmyr M, et al (2012) SOCS2 is dispensable for BCR/ABL1-induced chronic myeloid leukemia-like disease and for normal hematopoietic stem cell function. Leukemia (in press). 10.1038/leu.2012.169 PMC3542906 22824785

[41] Favre H, Benhamou A, Finidori J, Kelly PA, Edery M (1999) Dual effects of suppressor of cytokine signaling (SOCS-2) on growth hormone signal transduction. FEBS Lett 453: 63–66. 10403376 10.1016/s0014-5793(99)00681-x

[42] Tannahill GM, Elliott J, Barry AC, Hibbert L, Cacalano NA, et al (2005) SOCS2 can enhance interleukin-2 (IL-2) and IL-3 signaling by accelerating SOCS3 degradation. Mol Cell Biol 25: 9115–9126. 16199887 10.1128/MCB.25.20.9115-9126.2005 PMC1265772

[43] Greenhalgh CJ, Rico-Bautista E, Lorentzon M, Thaus AL, Morgan PO, et al (2005) SOCS2 negatively regulates growth hormone action in vitro and in vivo. J Clin Invest 115: 397–406. 15690087 10.1172/JCI22710 PMC546423

[44] Hookham MB, Elliott J, Suessmuth Y, Staerk J, Ward AC, et al (2007) The myeloproliferative disorder-associated JAK2 V617F mutant escapes negative regulation by suppressor of cytokine signaling 3. Blood 109: 4924–4929. 17317861 10.1182/blood-2006-08-039735

[45] Hosoya T, Maillard I, Engel JD (2010) From the cradle to the grave: activities of GATA-3 throughout T-cell development and differentiation. Immunol Rev 238: 110–125. 20969588 10.1111/j.1600-065X.2010.00954.x PMC2965564

[46] Sundrud MS, Grill SM, Ni D, Nagata K, Alkan SS, et al (2003) Genetic reprogramming of primary human T cells reveals functional plasticity in Th cell differentiation. J Immunol 171: 3542–3549. 14500650 10.4049/jimmunol.171.7.3542

[47] Abraham RM, Zhang Q, Odum N, Wasik MA (2011) The role of cytokine signaling in the pathogenesis of cutaneous T-cell lymphoma. Cancer Biol Ther 12. 10.4161/cbt.12.12.18144 PMC3679094 22236880

[48] Krejsgaard T, Ralfkiaer U, Clasen-Linde E, Eriksen KW, Kopp KL, et al (2011) Malignant cutaneous T-cell lymphoma cells express IL-17 utilizing the Jak3/Stat3 signaling pathway. J Invest Dermatol 131: 1331–1338. 21346774 10.1038/jid.2011.27

[49] Ellinghaus D, Ellinghaus E, Nair RP, Stuart PE, Esko T, et al (2012) Combined analysis of genome-wide association studies for Crohn disease and psoriasis identifies seven shared susceptibility loci. Am J Hum Genet 90: 636–647. 22482804 10.1016/j.ajhg.2012.02.020 PMC3322238

[50] Fridman JS, Scherle PA, Collins R, Burn T, Neilan CL, et al (2011) Preclinical evaluation of local JAK1 and JAK2 inhibition in cutaneous inflammation. J Invest Dermatol 131: 1838–1844. 21677670 10.1038/jid.2011.140

