# Supplementary figures and images for: t(8;9)(p22;p24)/PCM1-JAK2 Activates SOCS2 and SOCS3 via STAT5
Source: PLoS One. 2013 Jan 23;8(1):e53767. doi: 10.1371/journal.pone.0053767 (PMC3553112; doi:10.1371/journal.pone.0053767)

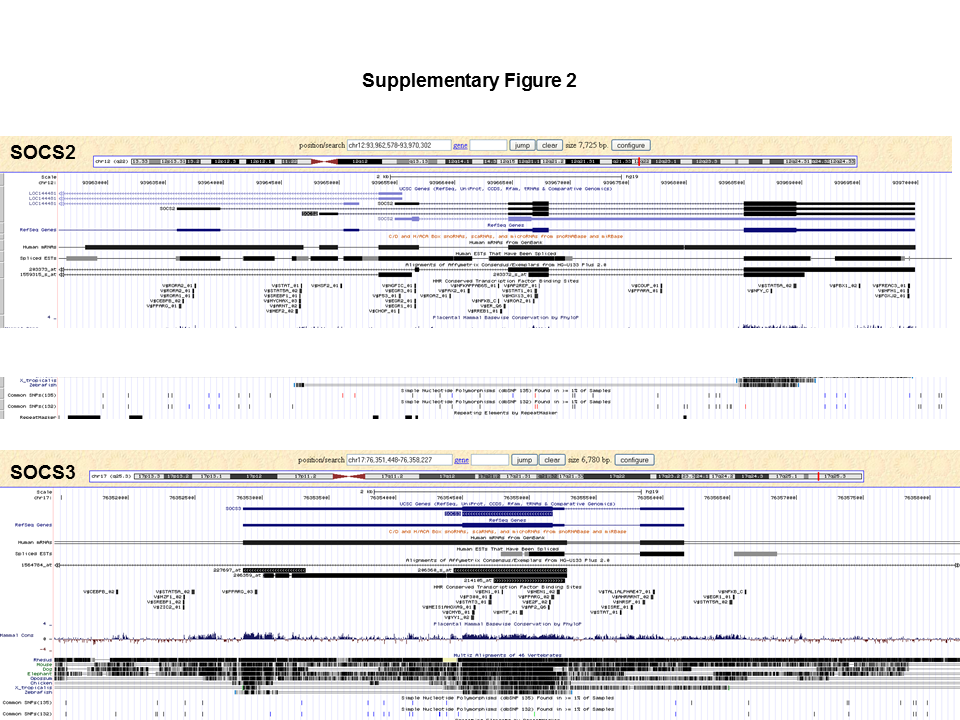

Supplement: Figure S2 — Transcription factor binding sites of STAT3 and STAT5. The figure is drawn from the UCSC browser (HG19) and covers SOCS2 and SOCS3. Note presence of TFBS for STAT3 and STAT5 at both SOCS loci. (TIF) [file pone.0053767.s002.tif]

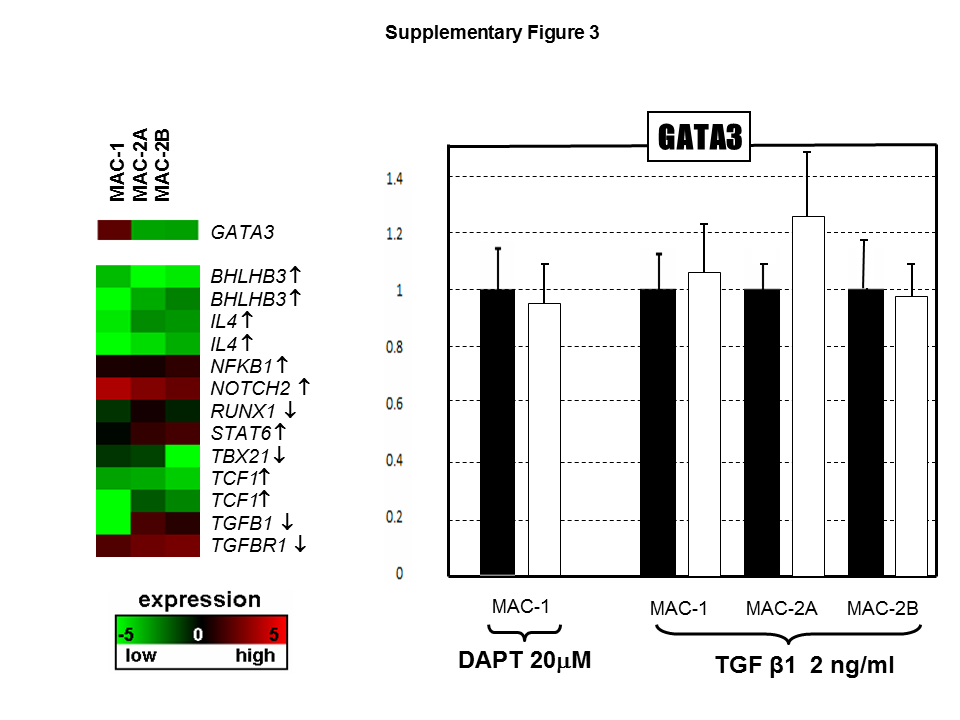

Supplement: Figure S3 — Differential expression of GATA3 regulators in MAC-1/2A/2B cells. Microarray heatmap (left) shows expression of known GATA3 regulators (arrows showing whether positive/negative). NOTCH2 (positive) and TGFβ1 (negative) regulators like GATA3 are differentially regulated in MAC-1 and MAC-2A/B cells. However, normalized GATA3 expression (RQ-PCR) after 72h treatment with neither DAPT (γ-secretase inhibitor) nor TGFβ1 evidence modulation (right). Vehicle control data (black) shown left of treatment. (TIF) [file pone.0053767.s003.tif]
