# Supplementary material for: t(8;9)(p22;p24)/PCM1-JAK2 Activates SOCS2 and SOCS3 via STAT5
Source: PLoS One. 2013 Jan 23;8(1):e53767. doi: 10.1371/journal.pone.0053767 (PMC3553112; doi:10.1371/journal.pone.0053767)
Supplement: Methods S1 — (DOCX) [file pone.0053767.s007.docx]

**SUPPORTING INFORMATION: METHODS**

**Construction of lentiviral vectors.** Lentiviral transgenic plasmids were constructed containing H1-shRNA expression cassettes located in the U3 region of the Δ3´-LTR (12). To generate the lentiviral plasmid pdcH1-*PCM1-JAK2*-1-SR the plasmid pH1-*PCM1-JAK2*-1 was digested with *Sma*I and *Hinc*II and the resulting DNA fragment (360 nt) blunt-end ligated into the *Sna*BI site of the pdc-SR. The lentiviral plasmids encode RFPEXPRESS as reporter gene. Primer sequences are available on request.

**Preparation of recombinant lentiviral supernatants and lentiviral transduction.** VSV.G-pseudotyped lentiviral particles were generated by calcium phosphate co-transfection of 293T cells. Viral supernatants were concentrated by low-speed centrifugation. dcH1-shRNA-SR lentiviral preparations were titrated in triplicate by serial dilutions of the concentrated vector stocks on 1 x 10^5^ cells in 24-well plates. The number of RFP positive cells was analysed 72 h post-transduction by flow-cytomety analysis (FACS-Calibur, Becton-Dickinson, Heidelberg/Germany), and the titers typically ranging between 1-5 x 10^8^ IU/ml. Lentiviral supernatants were used to transduce cells for silencing of *PCM1-JAK2* with an MOI between 2 and 4 as described previously (11).
